# Paradoxical Vocal Fold Dysfunction Mimicking Bronchial Asthma in a Young Female Patient

**DOI:** 10.7759/cureus.41376

**Published:** 2023-07-04

**Authors:** Raman Goit, Prakash Raj Oli, Quang Le, Amit Bhandari

**Affiliations:** 1 Internal Medicine, Kathmandu Medical College, Kathmandu, NPL; 2 Internal Medicine, Province Hospital, Surkhet, NPL; 3 Hospital Medicine, University of Missouri School of Medicine, Columbia, USA; 4 Internal Medicine, St John's Hospital, Springfield, USA

**Keywords:** gastroesophageal reflux disease (gerd), flexible laryngoscope, paradoxical vocal fold dysfunction, bronchial asthma, inducible laryngeal obstruction

## Abstract

Inducible laryngeal obstruction (ILO)/paradoxical vocal fold motion (PVFM) is a reversible narrowing of the larynx that results from vocal fold motion dysfunction. Distinguishing ILO from asthma can be challenging, as they can coexist, and standard tests may not be able to differentiate between the two. However, a flexible laryngoscopy can promptly diagnose ILO. Unfortunately, ILO is often overlooked as a potential cause when evaluating patients with sudden-onset breathing difficulties and respiratory distress. We present a case of a young female who sought frequent treatment at the emergency room (ER) with symptoms of shortness of breath (SOB), rapid heart rate, rapid breathing, and anxiety. Despite receiving treatment for acute asthma attacks, her symptoms persisted. During her most recent hospital admission, a flexible laryngoscopy revealed abnormal vocal fold movements, indicating paradoxical vocal fold dysfunction (PVFD) and muscle tension dysphonia (MTD). A comprehensive treatment approach involving speech therapy, pulmonology, and psychiatry led to significant clinical improvement. This case report highlights the importance of raising awareness among healthcare providers about ILO potentially mimicking bronchial asthma exacerbation.

## Introduction

Inducible laryngeal obstruction (ILO), also known as paradoxical vocal fold motion (PVFM), is a condition characterized by periodic reversible blockage of the larynx triggered by external factors like exercise, irritants, stress, and anxiety [1]. It is more common in young females [2] but can affect individuals of all ages. Distinguishing ILO from asthma can be difficult since they could share similar symptoms, and standard tests may not provide a clear distinction between the two [1]. Hence, to confirm the diagnosis, flexible laryngoscopy should be performed, which reveals abnormal adduction of the true vocal folds, and bunching of the false vocal folds during both inspiration and expiration phases of respiration [3]. We describe a case of a 21-year-old female who presented multiple times to the emergency room (ER) with shortness of breath (SOB), rapid breathing, and elevated heart rate. Despite receiving treatment for bronchial asthma, her symptoms persisted until she was ultimately diagnosed with ILO through flexible laryngoscopy. This report highlights the importance of educating clinicians about this condition, as it is often overlooked in patients presenting with acute respiratory distress and breathing difficulties.

## Case presentation

A 21-year-old woman with a medical history of asthma, gastroesophageal reflux disease (GERD), depression, and generalized anxiety disorder (GAD) presented to the ER with intermittent SOB, chest pain, diaphoresis, and tachycardia. Prior to this presentation, she had been seen nine times in the ER over the past three years with similar complaints. Her surgical history was significant for tonsillectomy and adenoidectomy. One day prior, she had presented to the ED with complaints of palpitation, lightheadedness, and SOB. En route to the ED, her heart rhythm had raised concerns for supraventricular tachycardia (SVT), for which she had received intravenous adenosine with no changes in the rhythm and heart rate. Electrocardiogram (EKG) in the ED had shown sinus tachycardia. Then, she had been treated with ondansetron for nausea and vomiting, potassium for hypokalemia, and nebulized for SOB. She had been subsequently discharged on a Holter monitoring device, lorazepam for panic attacks, and was advised to follow up at the cardiology clinic in two days. However, by the following day, she had developed chest pain, SOB, numbness, and tingling in her hands and feet and presented to another ED. She had been tachycardic, and her EKG had been concerning for SVT. She had received two doses of IV adenosine 6 mg and 12 mg but had shown no response. Following this, she had been subsequently transferred to our facility.

The patient was tachycardic on arrival with stable blood pressure. Her essential blood work and radiology reports were within the normal range (Table 1).

**Table 1:** Initial labs at the time of presentation/admission to our hospital *Outside the normal range CBC: complete blood count; MCV: mean corpuscular volume; BUN: blood urea nitrogen; ProBNP: pro-brain natriuretic peptide

Labs	Patient values	Reference range and units
CBC		
White cell count	11.88*	4.00 – 10.80 x 10^3^/uL
Hemoglobin	12	13.0 – 18.0 G/DL
Hematocrit	38.1	37% to 52%
MCV	92.5	78.0 – 100.0 FL
Platelets	358*	150 – 350 x 10^3^/uL
Comprehensive metabolic profile		
Sodium	139	136 – 145 MMOL/L
Potassium	4	3.5 – 5.1 MMOL/L
Chloride	113*	98 – 107 MMOL/L
Bicarbonate	20.7*	21.0 – 32.0 MMOL/L
Anion gap	5.3	5.0 – 15.0 MMOL/L
BUN	11	7 – 18 MG/DL
Creatinine	0.76	0.70 – 1.30 MG/DL
Glucose	98	74 – 106 MG/DL
Calcium	9.4	8.5 – 10.1 MG/DL
Total protein	7.7	6.4 – 8.2 G/DL
Total bilirubin	0.2	0.2 – 1.0 MG/DL
Alkaline phosphatase (ALP)	88	52 – 144 U/L
Aspartate transaminase (AST)	17	15 – 37 U/L
Alanine transaminase (ALT)	17	13 – 56 U/L
International normalized ratio (INR)	0.9	0.8-1.1
Cardiac profile		
ProBNP	22	<125 PG/ML
Troponins	<3	0 – 53 ng/L
Thyroid-stimulating hormone (TSH)	2.75	0.358 – 3.740 uIU/ML

CT angiography of the chest showed a 9 mm pleural-based nodule in the right middle lobe. A few hours later, she complained of worsening SOB, became tachycardic and tachypneic, and was in visible respiratory distress with drooling, and stridor. In light of the suspicion of anaphylaxis, emergent intubation was done, and mechanical ventilation was initiated for airway protection. During intubation, her vocal cords were found adducted, with no obvious vocal cord edema. She was treated with epinephrine twice, as well as Benadryl, famotidine, and steroids. She was admitted to the ICU and was subsequently extubated to room air within 10 hours with clinical improvement. Subsequently, an electrophysiology consultation was done. Her previous EKGs, rhythm strips along with Holter monitor recordings were reviewed. She was found to have sinus tachycardia without evidence of SVT. She was also seen by pulmonary service, and treated with steroids, and budesonide inhalers for asthma. She was planning for a methacholine challenge test as an outpatient after discharge.

Despite these efforts, she continued to have intermittent episodes of SOB, tachycardia, and stridor. She subsequently had a bedside flexible laryngoscopy, which revealed bilateral true vocal folds with paradoxical movement, abducting with attempted phonation, and adducting with inspiration, which suggested a diagnosis of paradoxical vocal fold dysfunction (PVFD) and muscle tension dysphonia (MTD). She was instructed to perform rescue breathing exercises at the bedside, which led to improvement in her symptoms. She was advised to continue with rescue and diaphragmatic breathing with an onset of acute vocal fold dysfunction. A referral was made for the continuation of speech therapy. She was also seen by a psychiatrist and started on sertraline for GAD. She was subsequently discharged on the seventh day of her admission with plans to continue speech therapy and psychotherapy and follow up with pulmonary and cardiology; the 30-day event monitor was retained at discharge. On her first follow-up visit after two weeks of discharge, she was doing well and reported no further episodes. A review of the Holter monitor showed no signs of SVT or any arrhythmia.

## Discussion

ILO/PVFD is a rare condition that can mimic asthma [1] and anaphylaxis [4] and is more common among athletes and adolescents who engage in vigorous exercise. Of note, 5-7% of adolescents and young adults were found to have exercise-induced intermittent laryngeal obstruction (EILO) in Northern Europe [5,6]. There is a relationship between EILO and congenital laryngomalacia (CLM), and an increase in laxity of muscles, ligaments, or laryngeal cartilages [1]. A descriptive study involving 94 asthmatics and 40 controls showed that ILO was present in 19% of asthma patients [7]. In one observational study, 169 patients with difficult-to-treat asthma with recurrent attacks were found to have ILO/PVFM. These patients showed dysfunctional breathing and preserved lung function [8]. A study among military personnel diagnosed with ILO showed a strong correlation between ILO and combat stress in a background of GERD, rhinitis, anxiety disorder, conversion disorder, major depression, and factitious disorder [9]. Bronchoprovocation testing with methacholine challenge could not differentiate between ILO and severe asthma overlapping with ILO [1]. Diagnosis of ILO is primarily done with laryngoscopy, which reveals abnormal adduction of the membranous true vocal folds and adduction or bunching of the false vocal folds [3]; however, chest imaging with a CT scan is necessary to rule out other causes of dyspnea. Dynamic CT imaging can help assess ILO directly in those who are unable to undergo a direct examination [10].

Our patient had been clinically diagnosed with asthma by her pediatrician, but she had never had provocative testing to establish this diagnosis. She had been treated with albuterol inhalers as needed. Despite this, she had had multiple ED visits with SOB, anxiety, tachycardia, and palpitation. Despite concerns for SVT, on multiple EKGs, rhythm monitoring, and treatment with adenosine, her Holter monitor, albeit of a short duration of one week, did not show evidence of SVT. She was even treated for possible angioedema based on her symptomatology; however, she had no swelling on her oral mucosa or vocal cords, which makes a diagnosis of angioedema highly unlikely. Furthermore, she continued to have symptoms despite being treated with epinephrine, steroids, and famotidine. A diagnosis of ILO/PVFD was confirmed with flexible laryngoscopy. It is unclear if her symptoms were due to a combination of ILO/PVDF and asthma. Planned bronchoprovocation testing as an outpatient will likely provide a more definitive answer to this question. This case highlights the need to educate clinicians on this rare condition and to consider laryngoscopy early in the course for prompt diagnosis and appropriate treatment to prevent frequent hospitalizations and avoid unnecessary treatments.

The primary goal in treating ILO is to alleviate acute airway obstruction and help patients breathe spontaneously. The mainstay of treatment is speech therapy, which incorporates breathing exercises such as nasal breathing, panting, diaphragmatic breathing, and increasing inspiratory muscle strength [1,11]. Some studies suggest that medications like ipratropium, low-dose tricyclic antidepressants, and selective serotonin reuptake inhibitors, when used in conjunction with speech and breathing exercises, may be effective, but further research is needed in this field [1]. For patients who do not respond to standard treatment, a surgical approach may be considered, particularly for those with supraglottic ILO. The procedure involves lowering the height of the aryepiglottic fold to widen the laryngeal inlet and reduce obstruction, thereby improving exercise ventilatory capacity [1]. A retrospective review of pediatric patients emphasized the importance of speech therapy, breathing exercises, and relaxation techniques in managing ILO, particularly in those with associated conditions like asthma, GERD, and allergies [12]. We followed a similar approach in the management of our patient. We observed the beneficial effects of speech therapy and breathing exercises in relieving her acute SOB. We employed a multidisciplinary approach involving a psychiatrist, pulmonologist, and cardiologist in managing our patient.

## Conclusions

ILO/PVFD, which can overlap with bronchial asthma, is a rare condition and often not at the forefront of differential diagnosis in patients who present with an acute-onset SOB. However, it is easily diagnosed at the bedside with a flexible laryngoscope and is treatable. Patients with a history of depression, anxiety, conversion disorder, asthma, or GERD presenting with SOB, anxiety, and tachycardia but no lower respiratory disease findings should undergo a prompt diagnostic flexible laryngoscopy. This enables early diagnosis of ILO, thereby facilitating the immediate application of breathing exercises and techniques alongside emergent medical management for prompt intervention.
